# Safety First! Residential Group Climate and Antisocial Behavior: A Multilevel Meta-analysis

**DOI:** 10.1177/0306624X241252052

**Published:** 2024-06-10

**Authors:** Eltink E. M. A, Roest J. J, Van der Helm G. H. P, Heynen E. J. E, Kuiper C. H. Z, Nijhof K. S, Vandevelde S, Leipoldt J. D, Stams G. J. J. M, Knorth E, Harder A.T, Assink M

**Affiliations:** 1 University of Amsterdam; GGZ Centraal, the Netherlands; 2 University of Applied Sciences Leiden, the Netherlands; 3 University of Amsterdam; University of Applied Sciences Leiden, the Netherlands; 4 Open University Netherlands, the Netherlands; 5 University of Amsterdam; University of Applied Sciences Leiden, the Netherlands; 6 Academic Workplace for at-risk Youth (AWRJ); Pluryn; Radboud University Nijmegen, the Netherlands; 7 Ghent University, Belgium; 8 University of Amsterdam, the Netherlands; 9 University of Groningen, the Netherlands; 10 Erasmus University Rotterdam, the Netherlands

**Keywords:** antisocial behavior, group climate, residential facility, meta-analysis, aggression, recidivism, youth, adult

## Abstract

A systematic review and multilevel meta-analysis was performed (28 studies and 313 effect sizes) on the relation between residential group climate (i.e., safety, atmosphere, repression, support, growth, structure) and antisocial behavior, including aggression and criminal recidivism. A systematic search was conducted in PsychINFO, ERIC, and OVID Medline up to February 2023. Results showed a small but significant association (*r* = .20) between residential group climate and antisocial behavior, equivalent to a 23% reduction of antisocial behavior in all clients receiving care in a residential facility with a therapeutic group climate. Moderator analyses showed that experienced safety was more strongly related to antisocial behavior (*r* = .30) than the other dimensions of group climate (.17 < *r* < .20), while the effect size was somewhat larger for adults (*r* = .24) than for youth (*r* = .15). We conclude that residential facilities should consider safety as a priority and should involve clients in a positive process of change through the development of a therapeutic environment and delivery of evidence-based treatment, addressing their needs from the perspective of rehabilitation.

## Introduction

Residential facilities that provide 24-hour therapeutic care include psychiatric hospitals, forensic residential facilities and open, semi-secure, and secure residential care facilities for youth and adults with complex or special needs, such as clients with emotional and behavioral disorders and/or intellectual disabilities, and individuals showing criminal behavior and/or substance abuse (Bowers et al., 2009; Dickens et al., 2014; Knotter et al., 2016; Leipoldt et al., 2019). Three meta-analyses showed that residential treatment may lead to positive outcomes for children (Strijbosch et al., 2015), adolescents (De Swart et al., 2012), and adults (Yoon et al., 2017). Another meta-analysis supported the overall effectiveness of prison-based therapeutic communities for adults (Lees et al., 2004). In addition, residential rehabilitation programs can decrease re-offending rates among formerly incarcerated offenders (see e.g., Lipsey, 2009; Pompoco et al., 2017; Van Stam et al., 2014).

Despite literature demonstrating positive effects of residential care, there is an ongoing discussion on the use and effectiveness of residential care for youth (Gutterswijk et al., 2020) and adults (Parhar et al., 2008), in particular because residential care deprives people of their freedom, and can result in repression by staff and antisocial behavior of residents (De Valk et al., 2019). Institutional repression hinders the three basic needs of self-determination (autonomy, relatedness, and competence) that are needed for individual growth, motivation, and positive development (De Valk et al., 2019; Van der Helm et al., 2018).

Many individuals who are placed in residential facilities have a history of antisocial behavior, defined as behavior that psychically or psychologically harms others or their property, which shows lack of consideration for the well-being of others, or violates the basic rights of others (Calkins & Keane, 2009; Stoff et al., 1997). Antisocial behavior emerges as aggression, delinquent behavior, and violence. It can be reinforced by a number of negative environmental influences that are associated with residential placement itself, such as repression, deprivation of meaningful relationships with important others, such as attachment figures or natural mentors (DeLisi et al., 2011; De Valk et al., 2019; Eltink et al., 2018; Van Dam et al., 2018). Moreover, antisocial behavior of clients in residential care can have a negative impact on the relationships among clients and client–staff relationships, which are core aspects of residential group climate (Schubert et al., 2012; Van der Helm, Boekee, et al., 2011).

### Residential Group Climate

The literature on residential group climate has a long history. Clemmer (1940, p. 279) introduced the term “prisonization,” which he described as the “taking on, in greater or lesser degree, of the mores, customs, and general culture of the penitentiary” by prisoners. Goffman (1961) considered residential facilities as total facilities, because all aspects of life take place within the residential facility. Activities follow a tight schedule and are imposed by formal rules to fulfill the aims of the facility, which results in a loss of the responsibilities of the residents, resulting in hospitalization (Goffman, 1961). The imposed structure ensures that residents are manageable and adapt themselves to the standards of the facility (Foucault & Mailänder, 1975). Individuals are pressured to conform (Foucault & Mailänder, 1975; Merton & Merton, 1968). However, the pioneers of group-based therapeutic care (Addams, 1910; Korczak, 1925/1992) rejected repressive regiments of discipline and control, and advocated care in their service delivery (Maier, 1987; Polsky et al., 1968).

The terminology to describe residential group climate is diverse (for an overview, see Tonkin, 2016), and ranges from “social climate” (e.g., Langdon et al., 2004; Schalast et al., 2008; Tonkin, 2016), “ward atmosphere” (Moos, 1975), “prison social climate” (Casey et al., 2016; Ross et al., 2008), “therapeutic residential care” (Leipoldt et al., 2019; Whittaker et al., 2015) to “(living) group climate” (Van der Helm et al., 2018).

Van der Helm et al. (2018) developed a definition of group climate that summarizes the different descriptions of group climate in scientific literature from the perspective of therapeutic quality of residential treatment and rehabilitation. They based their definition on Self Determination Theory (SDT; Ryan & Deci, 2000), which assumes that the social environment has an impact on human motivation by its effects on competence, relatedness with others, and possibilities to experience or execute personal autonomy. Van der Helm et al. (2018) defined residential group climate as:

The quality of the social- and physical environment in terms of the provision of sufficient and necessary conditions for physical and mental health, well-being, contact and personal growth of the residents, with respect for their human dignity and human rights as well as (if not restricted by judicial measures) their personal autonomy, aimed at recovery and successful participation in society. (p. 340).

Six dimensions of residential group climate emerge in scientific literature. Support is the extent to which staff is responsive to residents’ psychological needs (Leipoldt et al., 2019; Ross et al., 2008). Growth refers to opportunities for learning and development (Moos, 1975; Moos & Houts, 1968; Wright, 1985). Structure concerns a predictable and consistent institutional order, with clear rules and regulations, and adequate supervision (e.g., Langdon et al., 2004; Leipoldt et al., 2019; Pinchover & Attar-Schwartz, 2014). Safety is the degree to which residents are protected against harm, threat, danger, and bullying from fellow-residents (Leipoldt et al., 2019; Robinson et al., 2018; Ross et al., 2008; Wright, 1985). The “atmosphere” dimension concerns the degree to which the physical and social environment foster feelings of safety and trust among residents (Moos & Houts, 1968; Van der Helm, Stams, et al., 2011; Robinson et al., 2018). Repression, finally, has been defined as “a transactional process between youth and authority figures, characterized by an authority figure intentionally acting in a way that harms the youth, or by an authority figure unlawfully or arbitrarily depriving the youth of liberty or autonomy” (De Valk et al., 2016, p. 205).

Residential group climate can be considered as therapeutic if residents feel safe, repression is low or absent, structure and possibilities for (personal) growth are high, and staff–client relationships as well as relationships among clients themselves are supportive (Van der Helm et al., 2018). There is empirical evidence showing that a therapeutic group climate fosters (intrinsic) motivation in clients to work on a positive change, and reduces the risk of antisocial behavior by affecting positive outcomes in various domains (Leipoldt et al., 2019; Schubert et al., 2012; Van der Helm et al., 2018), such as empathy and emotional stability (Heynen et al., 2017; Van der Helm, Stams, Van der Stel, et al., 2012), quality of life (Leipoldt et al., 2019), and social information processing (Eltink et al., 2015).

### Residential Group Climate and Treatment Outcomes

Several studies examined factors that may explain a link between residential group climate and antisocial behavior. Heynen et al. (2017) and Van der Helm, Stams, Van der Stel, et al. (2012) found that group climate was associated with empathy in detained male (adolescent) offenders, which has been shown to be related to delinquent behavior (Van Langen et al., 2014). Van der Helm et al. (2014) showed that therapeutic group climate was positively associated with active coping and treatment motivation among detained juvenile delinquents; a positive longitudinal association between residential group climate and motivation of detained justice-involved adolescents was also found in Van der Helm et al. (2018). Also, Van der Helm, Stams, Van Genabeek, & Van der Laan (2012) showed that therapeutic group climate was positively associated with the Big Five personality factors openness and agreeableness, and buffered against aggression through its positive effect on emotional stability in juvenile incarcerated offenders. Finally, Eltink et al. (2015) showed that therapeutic group climate was negatively associated with aggressiveness-related deficits in social information processing in detained adolescent offenders.

Research on residential group climate is accumulating (Leipoldt et al., 2019; Schaftenaar et al., 2018; Tonkin, 2016; Whittaker et al., 2015). Therefore, Leipoldt et al. (2019) conducted a narrative review of the literature on outcomes of group (social) climate in therapeutic residential youth care in western countries. They found a positive association between therapeutic group climate and various outcomes. Effect sizes ranged from small to large, and showed heterogeneity within and between studies due to the variation in the concepts and operationalizations of group climate. It was concluded that residential youth care facilities should invest in a group climate that is supportive, structured, and caring, providing youth with an environment that enables growth.

Robinson et al. (2018) were the first to conduct a narrative review of the literature on group climate and aggression. They found that in most studies a therapeutic group climate was associated with less client aggression. Discrepancy in results were explained by differences in facilities, samples, group climate questionnaires, and measures of aggression. Robinson et al. (2018) concluded that in order to reduce aggressive behavior residential care facilities need to focus on supporting individual clients in managing their aggressive behavior as well as on establishing a therapeutic group climate.

### Present Study

Given the growing literature on residential group climate and accumulating empirical evidence for the relation between residential group climate, the aim of present study is to examine the relation between residential group climate and antisocial behavior by means of meta-analysis, and examine a number of factors possibly explaining this link. We included studies on youth and adults, because the literature on living group climate reveals that many commonalities exist in residential group care for youth and adults, while similar approaches in the assessment of group climate in residential facilities for youth and adults are applied.

The present meta-analysis is the first to compare the association between group climate and antisocial behavior in youth and adults. The present study is the first meta-analysis to quantitatively integrate the extant empirical literature on the relation between residential group climate and juvenile and adult antisocial behavior, that is, aggression and criminal offense recidivism, by examining the strength of this relation, accounting for possible moderating effects of study characteristics (e.g., study design, country), sample characteristics (e.g., age category, type of facility), climate characteristics (e.g., dimension, type of climate measure), and outcome characteristics (e.g., type of antisocial behavior and rater group climate/aggression).

## Method

### Selection of Studies

This meta-analysis was guided by the Preferred Reporting Items for Systematic Reviews and Meta-Analyses (PRISMA; Page et al., 2020). Studies were included in the meta-analysis if they met six criteria: (a) using quantitative measures of residential group climate and antisocial behavior, including aggression and delinquent behavior; (b) reporting on the bivariate association between group climate and antisocial behavior; (c) subjects live in a residential facility; and (d) results are published in a peer-reviewed journal. Single Case Experimental Design (SCED) and (quasi-)experimental studies were excluded, except when the necessary statistical information could be derived from pre-test assessments or a control group that did not receive intervention.

**Figure 1.**
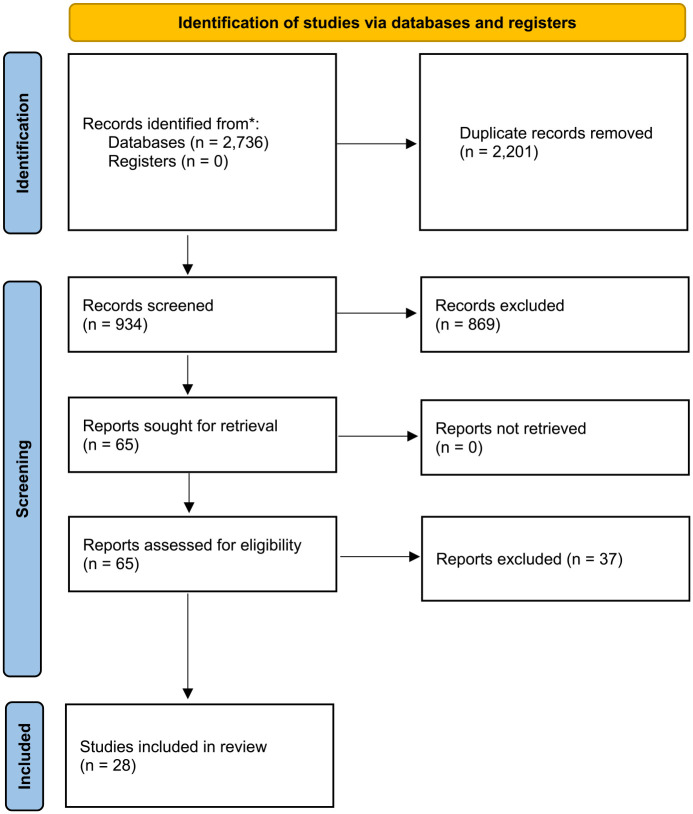
PRISMA flow chart of the search strategy and identification of studies.

Studies were collected until February 2023 by using multiple search methods. First, we searched for studies in the following electronic databases: PsychINFO, ERIC, and OVID Medline. Various terms related to social climate (e.g., group climate, living group climate, ward climate), externalizing behavior (e.g., aggres*, delinq*), and residential treatment were combined (see the appendix for the search string). Next, manual searches were conducted by inspecting reference lists of articles and reviews in order to find relevant studies that were not included yet. The search yielded 2,736 reports, of which 28 studies met the selection criteria (see Figure 1).

### Coding the Studies

A coding system was developed for moderators that could possibly affect the association between residential group climate and antisocial behavior, including publication, study design, sample, group climate, and outcome characteristics. Moderators were year of publication, journal impact factor, continent (North America or Europe), study design (cross-sectional or longitudinal), client age (youth or adults), type of facility (secure, semi-secure, open, mixed), gender (male, female, mixed), subjects (mixed, patients, intellectual disability, delinquents), group climate dimensions (support, safety, structure, growth, atmosphere, and institutional repression), group climate informant (client, staff, or composite score), group climate measure (EssenCES, PGCI, MQPL, WAS, or other), type of group climate score (individual score, group score), antisocial behavior (i.e., self-reported aggression, aggression incidents, criminal recidivism), and whether studies used client self-report ratings for both group climate and aggressive behavior (same rater, different rater). All 28 studies were coded by the first author. Eight studies were independently double coded by the first and second author, showing satisfactory inter-coder agreement (Cohen’s kappa .67–1.00). Discrepancies in coding were resolved through discussion between the authors. The study protocol was not previously registered.

All studies used well-validated instruments to assess both living group climate and antisocial behavior. Moreover, moderators were included to test effects of moderators that relate to study quality, such as study design, and the assessment of group climate and antisocial behavior. In addition, we rated the quality of studies by means of a tool that can be used for correlational studies (AXIS; Downes et al., 2016), which consisted of 20 items assessing aspects of clarity of research aims, quality of methods (e.g., validity of instruments, statistics) and results (e.g., response bias) as well as critical evaluation of research findings and outcomes (e.g., independence of researchers, study limitations, ethical approval). For each question that is answered positively, one point can be given, yielding a maximum score of 20 points. Since all studies were published in peer-reviewed journals, study quality was relatively high (*M* = 18.62; *SD* = 1.60). To test the impact of study quality, we added the study quality index as a moderator in the statistical analysis.

### Statistical Analyses

For each study, Pearson’s *r* was calculated as a measure for the association between residential group climate and antisocial behavior. Six studies reported on correlations between group climate and antisocial behavior at group level, based on averaged scores of climate and antisocial behavior. In these studies, the number of groups was used as the sample size of the study, since the group was the level of analysis. Effect sizes were coded in the expected direction, such that the overall effect size estimate indicated a positive relation between aspects of social climate and antisocial behavior.

Each correlation was transformed to Fisher’s *Z* before analyses, and back-transformed to *r* for the purpose of interpretation: *r* = .10 is a small effect, *r* = .25 is a medium effect, and *r* = .40 is a large effect (Cohen, 1988). For the purpose of clinical importance, we converted the overall effect size into a percentage of change using a formula provided by Kraemer and Kupfer (2006), transforming *r* into Area Under Curve (AUC), subsequently computing a percentage of change through: (2 * AUC – 1) * 100.

We checked for outliers by calculating standardized scores larger or smaller than 3.29 (Tabachnik & Fidell, 2013). Outliers were retained if they made no difference for the results. The homogeneity of the combined total effect size was tested with a Chi-Square test of both the within and between study variance. In the case of significant heterogeneity, moderators may account for within and between study differences, and moderator analyses should be conducted. Categorical variables were turned into dichotomous dummy codes, and continuous moderator variables were centered around their mean prior to conducting moderator analyses.

More than one effect size could be derived from most studies. We therefore used a three-level random effects model to account for dependency of effect sizes within studies (Van den Noortgate et al., 2014), with three sources of variance: sampling variance of the observed effect sizes (level 1), variance between effect sizes from the same study (level 2), and variance between studies (level 3).

We used the function “rma.mv” of the metafor package (Viechtbauer, 2010, 2015) in the R environment (version 3.5.2). The R syntax (Assink & Wibbelink, 2016) was based on procedures outlined by Van den Noortgate et al. (2014). The *t*-distribution was used for testing regression coefficients of the meta-analytic models, while categorical moderators consisting of three or more categories were tested by means of an omnibus *F*-test. To determine whether the variance between effect sizes from the same study (level 2), and the variance between studies (level 3) were significant, two separate one-tailed log-likelihood-ratio-tests were performed in which the deviance of the full model was compared to the deviance of a model excluding one of the variance parameters. All model parameters were estimated using the restricted maximum likelihood estimation method. The log-likelihood-ratio-tests were performed one-tailed, and all other tests were performed two-tailed. We considered *p*-values <.05 as statistically significant, and *p*-values <.10 as borderline-significant, or trends.

### Publication Bias/File Drawer Problem

Studies reporting significant associations are more likely to be published than studies with non-significant results, which can lead to an overestimation of the true effect size, referred to as the “file drawer problem” or “publication bias” (Rosenthal, 1979). We performed a “trim and fill procedure” (Duval & Tweedie, 2000), which tests whether effect sizes are missing on the left side of the distribution, indicating that the overall estimate found in the meta-analysis is an overestimation of the true effect. The trim and fill procedure could also indicate missing studies on the right side of the distribution, indicating that the overall estimate is an underestimation of the true effect.

We used the trim-and-fill procedure as outlined by Fernández-Castilla et al. (2021), which estimates the number of effect sizes imputed at the left and right side of the distribution to examine whether the overall effect size estimates were sensitive to potential presence of publication bias. Fernández-Castilla et al. (2021) employ a method in which the estimated number of effect sizes on the left side of the funnel plot distribution is related to a cutoff value of the estimator of the trim-and-fill method, based on the population ES and power (number of effect sizes). If the number of imputed studies exceeds the cutoff value at the left or right side of the funnel plot, this may be indicative of publication or selection bias, respectively.

Finally, we used Egger regression (Egger et al., 1997; Fernández-Castilla et al., 2021), which tests the degree of funnel plot asymmetry as measured by the intercept from regression of standard normal deviates (effect size divided by its standard error) against the estimate’s precision (the inverse of the standard error). A significant Egger regression test indicates funnel plot asymmetry.

## Results

### Descriptive Statistics of the Study Sample

The sample contained a total of 28 studies, including 313 effect sizes. The studies reporting on the residential group climate-antisocial behavior association included a total of *N* = 30,404 clients, and *N* = 1,321 staff; two of the studies only reported number of wards included (Bowers et al., 2009; Lanza et al., 1994). The mean sample size per study was 173 (*SD* = 233). Descriptives of the study sample are depicted in Table 1.

**Table 1.** Summary of Studies Included in the Meta-Analysis.

Study	Outcome	Instrument self-reported aggression	Instrument group climate	Population	Region	Informant group climate	*N*	Study design	Type facility	Effect size^b^
Auty and Liebling (2020)	Recidivism	—	MQPL	Adult	Europe	Client	24,508	Longitudinal	Closed, semi-closed	.23
Beijersbergen et al. (2015)	Aggression, Incidents	—	MQPL, DIS	Adult	Europe	Client	806	Longitudinal	Closed	.16
Beijersbergen et al. (2016)	Recidivism	—	MQPL, DIS	Adult	Europe	Client	1,241	Longitudinal	Closed	.09
Bowers et al. (2009)	Incidents	OAS	WAS	Adult	Europe	Staff	136^a^	Cross-sectional	Open	.27
De Decker et al. (2017)	Incidents	MOAS	PGCI	Youth	Europe	Client	24	Longitudinal	Semi-closed	.15
Dickens et al. (2014)	Incidents	OAS	EssenCES	Adult	Europe	Client	63	Cross-sectional	Semi-closed	.01
Eggert et al. (2014)	Incidents	—	EssenCES	Adult	North America	Staff	353	Cross-sectional	Closed, Semi-closed	.30
						Client	526			
Eltink et al. (2018)	Aggression	BDHI-D	PGCI	Youth	Europe	Client	198	Longitudinal	Open, closed, semi-closed	.18
Embregts et al. (2009)	Incidents	—	CAI	Adult	Europe	Staff	87	Cross-sectional	Open	.35
Heynen et al. (2017)	Aggression	RPQ	PGCI	Youth	Europe	Client	156	Cross-sectional	Closed	.05
Lanza et al. (1994)	Incidents	—	WAS	Adult	North America	Staff	6^a^	Cross-sectional	Open	.33
						Client				
Long et al. (2011)	Incidents	OAS	EssenCES	Adult	Europe	Staff	80	Cross-sectional	Semi	.26
						Client	65			
Mathys et al. (2013)	Aggression	QCGCR	QCGCR	Youth	North America	Client	153	Cross-sectional	Open	.15
Moos & Houts (1970)	Aggression	—	WAS	Adult	North America	Client	186	Cross-sectional	Open	.35
Neimeijer et al. (2021)	Incidents	—	PGCI	Adult	Europe	Client	248	Cross-sectional	Closed	.23
Puzzo et al. (2018)	Incidents	—	EssenCES	Adult	Europe	Staff	69	Cross-sectional	Closed, semi-closed	.21
						Client	42			
Reisig and Mesko (2009)	Aggression, Incidents	—	-	Adult	Europe	Client	103	Cross-sectional	Closed	.25
Robinson and Craig (2019)	Incidents	—	EssenCES	Adult	Europe	Client	13	Cross-sectional	Closed, semi-closed	.16
Ros et al. (2013)	Incidents	—	PGCI	Adult	Europe	Client	72	Longitudinal	Closed	.18
Schalast et al. (2008)	Incidents	—	EssenCES	Adult	Europe	Staff Client	333 327	Cross-sectional	Closed	.15
Scholte and Van der Ploeg (2000)	Incidents	YSR	—	Youth	Europe	Client	200	Longitudinal	Closed, semi-closed	.18
Schubert et al. (2012)	Recidivism	—	DoIE	Youth	North America	Client	519	Longitudinal	Closed	.02
Van den Tillaart et al. (2018)	Aggression & incidents	BDHI-D	PGCI	Youth	Europe	Client	159	Cross-sectional	Open, closed, semi-closed	.06
Tonkin et al. (2012)	Incidents	—	EssenCES	Adult	Europe	Staff Client	399 315	Cross-sectional	Closed, semi-closed	.19
Van der Helm, Stams, Van Genabeek, et al. (2012)	Aggression	BDHI-D	PGCI	Youth	Europe	Client	59	Cross-sectional	Closed	.19
Van der Helm et al. (2013)	Aggression	BDHI-D	PGCI	Youth	Europe	Client	128	Cross-sectional	Closed	.30
Van der Laan and Eichelsheim (2013)	Incidents	—	Survey	Youth	Europe	Client	207	Cross-sectional	Closed	.19
Van Wijk-Herbrink et al. (2021)	Incidents	—	PGCI	Youth	Europe	Client	86	Cross-sectional	Closed	.18

*Note*. BDHI-D = Burke Depression Hostility Index-Dutch; DoIE = Dimension Of Institutional Experience; MOAS = Modified Overt Aggression Scale; EssenCES = Essen Climate Evaluation Schema; OAS = Overt Aggression Scale; MQPL = Measurement of Quality of Prison Life; DIS = Dutch Inmate Survey; PGCI = Prison Group Climate Inventory; QCGCR = Questionnaire du Climat de Groupe en Centre de Réadaptation; RPQ = Reactive-Proactive Aggression Questionnaire; WAS = Ward Atmosphere Scale.

a Sample sizes are based on the number of groups.

b Mean effect sizes are based on the mean of all calculated effect sizes within a study.

### Relation Between Group Climate and Antisocial Behavior

**Figure 2.**
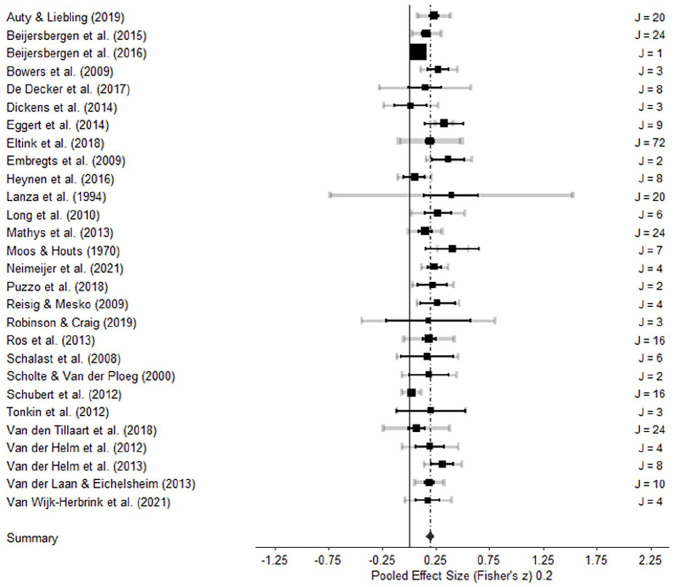
Forest plot. *Note*. The black line represents a confidence interval of the study’s effect size based on the sample size and total number of effect sizes within each study. The gray line represents an additional confidence interval based on the sampling variance of individual observed effect sizes within a study. The thickness of the gray confidence intervals is proportional to the number of effect sizes reported within studies. J = number of effect sizes.

The overall mean effect size between residential group climate and antisocial behavior was significant (*r* = .20, 95% CI = .16, .24, *p* < .001, *k* = 28, 313 ES), which indicates that a more therapeutic group climate was associated with less antisocial behavior, equivalent to a 23% reduction of antisocial behavior in clients receiving care in a residential facility with a therapeutic group climate. A forest plot of all studies is depicted in Figure 2. Nearly all studies demonstrated a positive overall effect in the expected direction, and the overall ES of 19 studies significantly deviated from zero, based on the 95% confidence interval.

**Figure 3.**
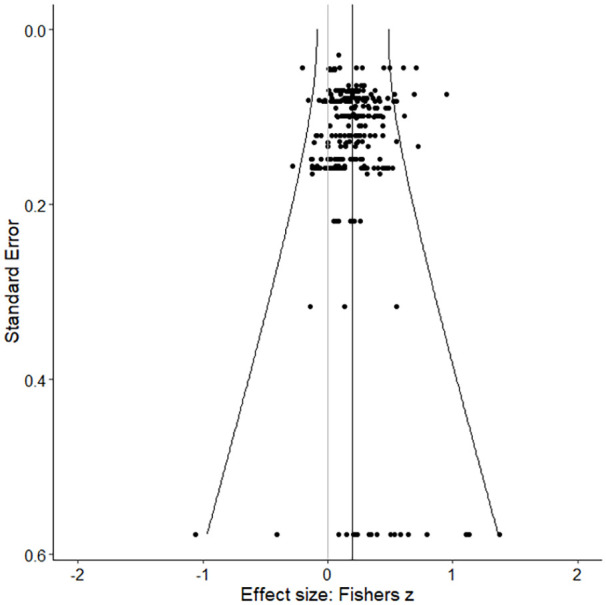
Funnel plot of all effect sizes.

**Figure 4.**
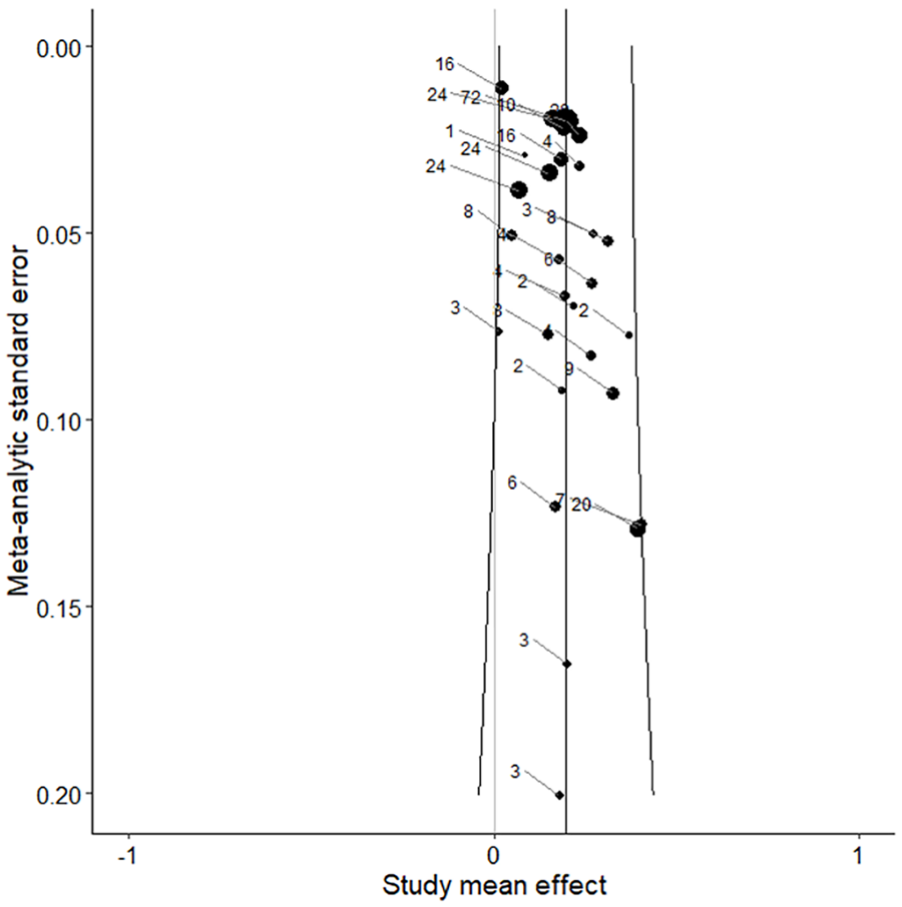
Funnel plot of study effects.

To assess the presence of publication bias a trim-and-fill procedure as well as a funnel plot and Egger’s test were performed. Results of the trim-and-fill procedure did not yield effect sizes that needed to be imputed at either the left or right side of the funnel plot in order to restore symmetry. The funnel plots for all effect sizes (Figure 3) and study effects (Figure 4) indicated symmetry, suggesting that there was no indication of publication bias. The results of the Funnel plot test (*z* = −0.19, *p* = .853) and Egger test (*z* = −0.29, *p* = .773) showed no significant effect, which also implies that there was no indication of publication bias.

Results further revealed substantial heterogeneity both within (51.4%) and between (22.9%) studies, which indicates that moderators could possibly impact the association between group climate and antisocial behavior. Therefore, moderator analyses were conducted.

### Moderator Analyses

All dimensions of group climate proved to be significant, but moderator analyses yielded a stronger effect for Safety (*r* = .30) than the other group climate dimensions (.17 < *r* < .20): *F*(5,304) = 2.482, *p* < .05. Also, a significant effect was found for publication year, indicating that more recent studies yielded smaller effects: *b* = −0.01, *t* = −2.759, *p* < .01. A significant effect was also found for age, indicating that the relation between group climate and antisocial behavior was stronger for adults (*r* = .24) than for youth (*r* = .15): *F*(1, 311) = 6.429, *p* < .05. A trend was found for study design. Longitudinal studies showed a somewhat smaller effect (*r* = .16) than cross-sectional studies (*r* = .21): *F*(1, 311) = 2.739, *p* < .10. Type of instrument showed a trend indicating differences in effect sizes between instruments: *F*(4, 308) = 2.130, *p* < .10. The relation between group climate and antisocial behavior was stronger when measured with the WAS (*r* = .35) compared to other measures (.17 < *r* < .22). Results of all moderator analyses are depicted in Table 2.

**Table 2.** Results of Moderator Analyses for the Association Between Group Climate and Antisocial Behavior.

Moderator	*k*	# ES	β_0_ *r*	*t*_0_	β_1_	*t*_1_	*F*(*df*_1_, *df*_2_)
Study characteristics							
Impact factor	28	313	.19	9.753***	.03	1.518	*F*(1, 311) = 2.305
Publication year	28	313	.19	10.423***	−01	−2.759	*F*(1, 311) = 7.614***
Region							*F*(1, 311) = 0.637
Europe (RC)	23	237	.19	8.077***			
United States	5	76	.23	5.126***	.04	0.798	
Design							*F*(1, 311) = 2.739+
Cross-sectional (RC)	23	222	.21	9.975***			
Longitudinal	8	91	.16	5.590***	−.05	−1.655+	
Quality index	28	313	.19	9.175***	−.01	−1.055	*F*(1, 311) = 1.113
Sample characteristics							
Age category							*F*(1, 311) = 6.4293*
Youth (RC)	11	180	.15	5.512***			
Adults	17	133	.24	9.592***	.09	2.536*	
Gender							*F*(2, 310) = 0.239
Mixed (RC)	22	264	.20	8.705***			
Female	2	30	.19	2.827**	−.01	−0.072	
Male	4	19	.16	2.552*	−.05	−0.691	
Subjects							*F*(3, 309) = 0.679
Mixed (RC)	3	13	.17	2.210*			
Patients	10	146	.21	7.449***	.04	0.458	
ID	3	9	.26	3.376***	.09	0.838	
Delinquents	14	145	.18	7.078***	.01	0.066	
Type of facility							*F*(3, 309) = 0.977
Closed (RC)	13	131	.17	6.660***			
Open	7	88	.19	5.658***	.02	0.474	
Semi-closed	5	49	.22	6.271***	.05	1.439	
Mixed	7	45	.23	5.192***	.06	1.127	
Climate characteristics							
Dimension							*F*(5, 307) = 2.466*
Safety (RC)	10	20	.30	7.774***			
Support	23	74	.19	7.115***	−.12	−2.885***	
Growth	15	58	.17	5.496***	−.14	−3.114***	
Repression	17	83	.20	7.670***	−.10	−2.528*	
Atmosphere	23	59	.17	5.694***	−.13	−3.173***	
Structure	7	19	.17	3.746***	−.13	−2.425*	
Type of climate measure							*F*(4, 308) = 2.130+
WAS (RC)	3	30	.35	5.726***			
MQPL	3	45	.18	3.588***	−.18	−2.206*	
PGCI	9	148	.17	5.648***	−.19	−2.712***	
ESSEN-CES	7	31	.22	4.965***	−.14	−1.835+	
Other	7	59	.17	5.407***	−.20	−2.657***	
Type of climate report							*F*(2, 310) = 1.471
Staff report (RC)	3	14	.30	3.625***			
Client report	22	275	.18	8.285***	−.12	−1.439	
Composite score	5	24	.24	4.774***	−.07	−0.677	
Type of climate score							*F*(1, 311) = 0.517
Individual scores (RC)	22	258	.19	8.466***			
Group scores	6	55	.23	4.740***	.04	0.719	
Outcome characteristics							
Type of antisocial behavior							*F*(2, 310) = 1.219
Self-reported aggression (RC)	10	147	.23	7.750***			
Aggression incidents	18	137	.18	6.806***	−.05	−1.449	
Criminal recidivism	3	29	.19	3.683***	−.04	−0.861	
Group climate/antisocial behavior							*F*(1, 311) = 2.597
Same rater (RC)	11	175	.23	7.982***			
Different rater	20	156	.18	7.475***	−.05	−1.612	

*Note. k* = number of independent studies; ES **=** effect size; RC = reference category.

* *p* < .05. **p < .01 ***p < .001.

### Multiple Regression Analysis

All borderline significant and significant moderators were entered in a multiple regression analysis in order to examine which moderators were unique. Results indicated a significant overall model: (*F*(5,307) = 5.465, *p* < .0001). Safety proved to be a significant and unique predictor (*b* = .12, *t* = 3.20, *p* < .0015), while age emerged as a borderline significant unique effect (*p* = .082).

## Discussion

The purpose of the present meta-analysis was to examine the relation between residential group climate and antisocial behavior of youth and adults. Results showed a small but significant association between residential group climate and antisocial behavior of *r* = .20, such that positive group climate was associated with less antisocial behavior, equivalent to a 23% reduction of antisocial behavior in clients receiving care in a residential facility with a therapeutic group climate. Moderator analyses showed that experienced safety was stronger related to antisocial behavior than the other dimensions of residential group climate, showing a significant and medium effect size of *r* = .30, which amounts to a 34% reduction of antisocial behavior. Furthermore, the association for youth (*r* = .15) was somewhat smaller than for adults (*r* = .24). Finally, more recent studies showed smaller effect sizes, but this effect was no longer significant after controlling for other significant or borderline-significant moderators.

A first explanation for the stronger effect of experienced safety may reside in its direct psychological survival value, since a threat to safety or security can substantially increase both stress levels and aggression (Moore et al., 2017, 2020; Willis & Willis, 2020). Another explanation for the finding that Safety correlates stronger with antisocial behavior than the other group climate dimensions is a contamination of the measures for safety and antisocial behavior, to the extent that assessments of unsafety and antisocial behavior both tap aggressive and violent behavior at the living group. The link between safety and antisocial behavior can also be understood as feeling safe in interactions with others, thus making a distinction between physical safety (including perceived threat to physical safety) and emotional safety (Gilbert, 2015). Experiencing safety is an important condition for successful therapy and healing (Bath, 2015). Furthermore, experiencing safety has a positive relation with social interaction with youth in a residential institution (Van der Laan & Eichelsheim, 2013). The more positive youth experience their interactions with peers and staff, the more safe they feel in an institution (Moore et al., 2017). Support from fellow clients can protect against unsafe situations, because clients can help each other survive dangerous situations, which enhances feelings of safety (Moore et al., 2020; O’Donnell & Edgar, 1999). This also shows that peers influence youths, and protection against antisocial behavior from others is important in a residential institution.

Finally, the association between residential group climate and antisocial behavior proved to be somewhat stronger in adults than in youth. One explanation for this result may be found in a study by Spruit et al. (2017), showing that dynamic (i.e., changeable) risk and protective factors for antisocial behavior, which are targets for judicial interventions, were more strongly related to criminal recidivism in adults than in youth. It is therefore plausible to suggest that adults are more susceptible to both negative and positive aspects of the residential therapeutic group environment than youth.

Another explanation may be found in the resilience of youth, who possibly can adjust better to a suboptimal group atmosphere than adults (Lucas et al., 2014). Youth may be more capable of handling a non-therapeutic group climate because of their greater resilience. Although adverse childhood experiences have been shown to be negatively associated with resilience in youth, a significant group of youth still grow up to be healthy adults (Ungar, 2013), in particular if they have supportive relationships with caregivers, and heightened sensitivity to rewards and positive stimuli (McLaughlin & Lambert, 2017), which emphasizes the importance of a therapeutic living group climate.

The overall small association between residential group climate and antisocial behavior of clients is an important finding, because antisocial behavior has been shown to be rather stable in detained adolescents (Eltink et al., 2018; Tremblay, 2010), which may be difficult to change due to a relatively strong genetic basis of antisocial behavior (Niv et al., 2013), low genetic susceptibility to (positive) environmental influences (Van IJzendoorn, & Bakermans-Kranenburg, 2015), and the accumulation of risk factors for antisocial behavior (Assink et al., 2015). Notably, it is unlikely that residential group climate alone could have positive effects on antisocial behavior given the multi-causal determination of antisocial behavior, which probably needs intensive evidence-based treatment to change (Andrews & Bonta, 2010).

The modest association between group climate and antisocial behavior might be spurious, that is, affected by a third unmeasured variable, such as evidence-based treatment that reduces antisocial behavior and goes together with a therapeutic group climate, although health service utilization in detained youth (White et al., 2019) and adults (Persson et al., 2017) has been shown to be low, and residential judicial interventions for youth have been shown to produce only small effects on criminal recidivism (Pappas & Dent, 2021). An example of evidence-based treatment that may successfully reduce antisocial behavior is Responsive Aggression Regulation Therapy (Re-ART; Hoogsteder et al., 2018). Other factors that may affect the relation between group climate and aggression are working climate, scale of the facility, and policies regarding aggression and seclusion (Molleman & Van Ginneken, 2015; Van Gink et al., 2018). Notably, well-trained staff may have a positive effect on both residential group climate and reduction of antisocial behavior, (partly) explaining the association between group climate and antisocial behavior. Given the empirical association between living group climate and antisocial behavior, policy makers should focus on the necessary conditions to achieve a therapeutic group climate, allowing for the rehabilitation of clients, patients, and offenders in residential facilities.

To conclude, only results from (quasi-)experimental research can prove whether residential group climate is causally related to antisocial behavior. For instance, Barton and Mackin (2012) and Schaftenaar et al. (2018) carried out quasi-experimental research, showing that group climate did positively affect criminal recidivism in youth and adult offenders.

Our meta-analysis has several limitations. A general limitation of the present meta-analysis is that it explains only a small part of the differences within and between studies by means of moderator analyses. To preserve a minimum level of study quality we only included published studies, which could increase the risk for publication bias. However, we used state-of-the-art three-level publication bias analyses, showing there was no indication of publication bias. A large number of potential moderators could not be tested due to insufficient information in the studies. Limited information on the residential facilities was provided, such as staff-resident ratio, group size, level of security, length of stay, information on same or mixed gender groups, physical design of the environment, education level and quality of staff, organizational management, group working methods, treatment delivery, treatment integrity, and whether treatments were evidence-based. These and other factors may explain heterogeneity in effect sizes within and between studies. Finally, no studies were available that “measure” group climate by means of observation instead of perception. Nevertheless, most studies used well-validated assessment instruments to assess both group climate and antisocial behavior, while no indication was found of same-reporter bias in moderator analysis, which is indicative of study quality. Some lower limit was achieved by only including articles that were published in peer-reviewed journals. Although the advantage of including unpublished studies may lie in increased clinical representativeness of study findings, we want to emphasize that most studies were carried out under clinically representative conditions.

## Conclusion

The present meta-analysis revealed that the association between residential group climate and antisocial behavior was moderate. Future research should test the possible causal influence of group climate on antisocial behavior through (quasi-)experimental research, and examine group climate as a moderator of treatment effectiveness. Future research should also assess more aspects that could influence the relation between residential group climate and antisocial behavior. For now, residential facilities should consider Safety as a priority by establishing a therapeutic environment, in combination with the delivery of evidence-based treatment that is responsive to the needs of clients from the perspective of rehabilitation.

## Supplemental Material

sj-docx-1-ijo-10.1177_0306624X241252052 – Supplemental material for Safety First! Residential Group Climate and Antisocial Behavior: A Multilevel Meta-analysis Supplemental material, sj-docx-1-ijo-10.1177_0306624X241252052 for Safety First! Residential Group Climate and Antisocial Behavior: A Multilevel Meta-analysis by Eltink E. M. A, Roest J. J, Van der Helm G. H. P, Heynen E. J. E, Kuiper C. H. Z, Nijhof K. S, Vandevelde S, Leipoldt J. D, Stams G. J. J. M, Knorth E, Harder A.T and Assink M in International Journal of Offender Therapy and Comparative Criminology

## References

[bibr1-0306624X241252052] Studies included in the meta-analysis are denoted with an asterisk (*)

[bibr2-0306624X241252052] Addams J. (1910). Twenty years at Hull House. Macmillan.

[bibr3-0306624X241252052] Andrews D. A. Bonta J. (Eds.). (2010). The psychology of criminal conduct (6th ed.). Routledge.

[bibr4-0306624X241252052] Assink M. Van der Put C. E. Hoeve M. De Vries S. L. A. Stams G. J. J. M. Oort F. J. (2015). Risk factors for persistent delinquent behavior among juveniles: A meta-analytic review. Clinical Psychology Review, 42, 47–61. 10.1016/j.cpr.2015.08.002 26301752

[bibr5-0306624X241252052] Assink M. Wibbelink C. M. (2016). Fitting three-level meta-analytic models in R: A step-by-step tutorial. The Quantitative Methods for Psychology, 12(3), 154–174. 10.20982/tqmp.12.3.p154

[bibr6-0306624X241252052] *Auty K. Liebling A. (2020). Exploring the relationship between prison social climate and reoffending. Justice Quarterly, 37(2), 358–381. 10.1080/07418825.2018.1538421

[bibr7-0306624X241252052] Barton W. Mackin J. (2012). Towards a strength-based juvenile correctional facility: Sustainability and effects of an institutional transformation. Journal of Offender Rehabilitation, 51(7), 435–452. 10.1080/10509674.2012.700688

[bibr8-0306624X241252052] Bath H. (2015). The three pillars of traumawise care: Healing in the other 23 hours. Reclaiming Children and Youth, 23(4), 5.

[bibr9-0306624X241252052] *Beijersbergen K. Dirkzwager A. Eichelsheim V. Van Der Laan P. Nieuwbeerta P. (2015). Procedural justice, anger, and prisoners’ misconduct: A longitudinal study. Criminal Justice and Behavior, 42(2), 196–218. 10.1177/0093854814550710 24009140

[bibr10-0306624X241252052] *Beijersbergen K. Dirkzwager A. Nieuwbeerta P. Holtfreter K. (2016). Reoffending after release: Does procedural justice during imprisonment matter? Criminal Justice and Behavior, 43(1), 63–82. 10.1177/0093854815609643

[bibr11-0306624X241252052] *Bowers L. Allan T. Simpson A. Jones J. Van der Merwe M. Jeffery D. (2009). Identifying key factors associated with aggression on acute inpatient psychiatric wards. Issues in Mental Health Nursing, 30(4), 260–271. 10.1080/01612840802710829 19363731

[bibr12-0306624X241252052] Calkins S. D. Keane S. P. (2009). Developmental origins of early antisocial behavior. Development and Psychopathology, 21(4), 1095–1109. 10.1017/S095457940999006X 19825259 PMC2782636

[bibr13-0306624X241252052] Casey S. Day A. Reynolds J. (2016). The influence of incarceration length and protection status on perceptions of prison social climate. Criminal Justice and Behavior, 43(2), 285–296. 10.1177/0093854815603747

[bibr14-0306624X241252052] Clemmer D. (1940). The prison community. Christopher.

[bibr15-0306624X241252052] Cohen J. (1988). Statistical power analysis for the behavioral sciences (2nd ed.). Erlbaum.

[bibr16-0306624X241252052] *De Decker A. Lemmens L. Van der Helm G. H. P. Bruckers L. Molenberghs G. Tremmery B. (2017). The relation between aggression and the living group climate in a forensic treatment unit for adolescents: A pilot study. International Journal of Offender Therapy and Comparative Criminology, 62, 1821–1837. 10.1177/0306624X17712347 28627274

[bibr17-0306624X241252052] DeLisi M. Trulson C. R. Marquart J. W. Drury A. J. Kosloski A. E. (2011). Inside the prison black box: Toward a life course importation model of inmate behavior. International Journal of Offender Therapy and Comparative Criminology, 55(8), 1186–1207. 10.1177/0306624X10383956 22114166

[bibr18-0306624X241252052] De Swart J. J. W. Van den Broek H. Stams G. J. J. M. Asscher J. J. Van der Laan P. H. Holsbrink-Engels G. A. Van der Helm G. H. P. (2012). The effectiveness of institutional youth care over the past three decades: A meta-analysis. Children and Youth Services Review, 34, 1818–1824. 10.1016/j.childyouth.2012.05.015

[bibr19-0306624X241252052] De Valk S. Kuiper C. H. Z. Van der Helm G. H. P. Maas A. J. J. A. Stams G. J. J. M . (2016). Repression in residential youth care: A scoping review. Adolescent Research Review, 1(3), 195–216. https://10.1007/s40894-016-0029-9

[bibr20-0306624X241252052] De Valk S. Kuiper C. H. Z. Van der Helm G. H. P. Maas A. J. J. A. Stams G. J. J. M . (2019). Repression in residential youth care: A qualitative study examining the experiences of adolescents in open, secure and forensic institutions. Journal of Adolescent Research, 34(6), 757–782. 10.1177/0743558417719188

[bibr21-0306624X241252052] *Dickens G. L. Suesse M. Snyman P. Picchioni M. (2014). Associations between ward climate and patient characteristics in a secure forensic mental health service. The Journal of Forensic Psychiatry & Psychology, 25(2), 195–211. 10.1016/j.apnu.2015.12.007

[bibr22-0306624X241252052] Downes M. J. Brennan M. L. Williams H. C. Dean R. S. (2016). Development of a critical appraisal tool to assess the quality of cross-sectional studies (AXIS). BMJ Open, 6(12), e011458. 10.1136/bmjopen-2016-011458 PMC5168618 27932337

[bibr23-0306624X241252052] Duval S. Tweedie R. (2000). Trim and fill: A simple funnel-plot-based method of testing and adjusting for publication bias in meta-analysis. Biometrics, 56, 455–463. 10.1111/j.0006-341x.2000.00455.x 10877304

[bibr24-0306624X241252052] Egger M. Davey Smith G. Schneider M. Minder C. (1997). Bias in meta-analysis detected by a simple, graphical test. BMJ, 315(7109), 629–634. 10.1136/bmj.315.7109.62 9310563 PMC2127453

[bibr25-0306624X241252052] *Eggert J. Kelly S. Margiotta D. Hegvik D. Vaher K. Kaya R. (2014). Person–environment interaction in a new secure forensic state psychiatric hospital. Behavioral Sciences & the Law, 32(4), 527–538. 10.1002/bsl.2127 25043717

[bibr26-0306624X241252052] *Eltink E. M. A. Ten Hoeve J. De Jongh T. Van der Helm G. H. P. Wissink I. B. Stams G. J. J. M . (2018). Stability and change of adolescents’ aggressive behavior in residential youth care, Child & Youth Care Forum, 47, 199–217. 10.1007/s10566-017-9425-y 29527107 PMC5834580

[bibr27-0306624X241252052] Eltink E. M. A. Van der Helm G. H. P. Wissink I. B. Stams G. J. J. M . (2015). “I stabbed him because he looked mean at me”. The relation between living group climate and reactions to social problem situations in detained adolescents. International Journal of Forensic Mental Health, 14, 101–109. 10.1080/14999013.2015.1033110

[bibr28-0306624X241252052] *Embregts P. J. C. M. Didden R. Huitink C. Schreuder N. (2009). Contextual variables affecting aggressive behaviour in individuals with mild to borderline intellectual disabilities who live in a residential facility. Journal of Intellectual Disability Research, 53(3), 255–264. 10.1111/j.1365-2788.2008.01132.x 19178616

[bibr29-0306624X241252052] Fernández-Castilla B. Declercq L. Jamshidi L. Beretvas S. N. Onghena P. Van den Noortgate W. (2021). Detecting selection bias in meta-analyses with multiple outcomes: A simulation study. The Journal of Experimental Education, 89(1), 125–144. 10.1080/00220973.2019.1582470

[bibr30-0306624X241252052] Foucault M. Mailänder E. (1975). Surveiller et punir (Vol. 225). Editions Gallimard.

[bibr31-0306624X241252052] Gilbert P. (2015). An evolutionary approach to emotion in mental health with a focus on affiliative emotions. Emotion Review, 7(3), 230–237.

[bibr32-0306624X241252052] Goffman E. (1961). Asylums: Essays on the social situation of mental patients and other inmates. Dubledzay. 10.1192/bjp.bp.113.140442 25587580

[bibr33-0306624X241252052] Gutterswijk R. V. Kuiper C. H. Z. Lautan N. Kunst E. G. Van der Horst F. C. Stams G. J. J. M. Prinzie P. (2020). The outcome of non-residential youth care compared to residential youth care: A multilevel meta-analysis. Children and Youth Services Review, 113, 104950. 10.1016/j.childyouth.2020.104950

[bibr34-0306624X241252052] *Heynen E. Van der Helm G. H. P. Cima M. Stams G. J. J. M. Korebrits A. (2017). The relation between living group climate, aggression, and callous-unemotional traits in delinquent boys in detention. International Journal of Offender Therapy and Comparative Criminology, 61(15), 1701–1718. 10.1177/0306624X16630543 26873150

[bibr35-0306624X241252052] Hoogsteder L. Stams G. J. J. M. Schippers E. E. Bonnes D. (2018). Responsive aggression regulation therapy (Re-ART): An evaluation study in a Dutch juvenile justice institution in terms of recidivism. International Journal of Offender Therapy and Comparative Criminology, 62, 4403–4424. 10.1177/0306624X18761267 29504484

[bibr36-0306624X241252052] Knotter M. H. Stams G. J. J. M. Moonen X. M. H. Wissink I. B. (2016). Correlates of direct care staffs’ attitudes towards aggression of clients with intellectual disabilities. Research in Developmental Disabilities, 59, 294–305. 10.1016/j.ridd.2016.09.008 27665412

[bibr37-0306624X241252052] Korczak J. (1992). When I am little again and the child’s right to respect. University Press of America. (Original work published 1925)

[bibr38-0306624X241252052] Kraemer C. Kupfer D. J. (2006). Size of treatment effects and their importance to clinical research and practice. Biological Psychiatry, 59(11), 990–996. 10.1016/j.biopsych.2005.09.014 16368078

[bibr39-0306624X241252052] Langdon P. E. Cosgrave N. Tranah T. (2004). Social climate within an adolescent medium-secure facility. International Journal of Offender Therapy and Comparative Criminology, 48(4), 504–515. 10.1177/0306624X03261559 15245660

[bibr40-0306624X241252052] *Lanza M. L. Kayne H. L. Hicks C. Milner J. (1994). Environmental characteristics related to patient assault. Issues in Mental Health Nursing, 15(3), 319–335. 10.3109/01612849409009393 7829320

[bibr41-0306624X241252052] Lees J. Manning N. Rawlings B. (2004). A culture of enquiry: Research evidence and the therapeutic community. Psychiatric Quarterly, 75(3), 279–294. 10.1023/b:psaq.0000031797.74295.f8 15335230

[bibr42-0306624X241252052] Leipoldt J. D. Harder A. T. Kayed N. S. Grietens H. Rimehaug T. (2019). Determinants and outcomes of social climate in therapeutic residential youth care: A systematic review. Children and Youth Services Review, 99, 429–440. 10.1016/j.childyouth.2019.02.010

[bibr43-0306624X241252052] Lipsey M. W. (2009). The primary factors that characterize effective interventions with juvenile offenders: A meta-analytic overview. Victims and Offenders, 4(2), 124–147. 10.1080/15564880802612573

[bibr44-0306624X241252052] *Long C. G. Anagnostakis K. Fox E. Silaule P. Somers J. West R. Webster A. (2011). Social climate along the pathway of care in women’s secure mental health service: Variation with level of security, patient motivation, therapeutic alliance and level of disturbance. Criminal Behaviour and Mental Health, 21(3), 202–214. 10.1002/cbm.791 21706527

[bibr45-0306624X241252052] Lucas C. G. Bridgers S. Griffiths T. L. Gopnik A. (2014). When children are better (or at least more open-minded) learners than adults: Developmental differences in learning the forms of causal relationships. Cognition, 131(2), 284–299. 10.1016/j.cognition.2013.12.010 24566007

[bibr46-0306624X241252052] Maier H. W. (1987). Developmental group care of children and youth: Concepts and practice. Routledge.

[bibr47-0306624X241252052] *Mathys C. Lanctot N. Touchette L. (2013). Construct validity and confirmatory factor analysis of a group climate measure for girls in residential care service: Real life intervention through three major dimensions. European Review of Applied Psychology/Revue Européenne de Psychologie Appliquée, 63, 109–119 10.1016/j.erap.2012.11.001

[bibr48-0306624X241252052] McLaughlin K. A. Lambert H. K. (2017). Child trauma exposure and psychopathology: Mechanisms of risk and resilience. Current Opinion in Psychology, 14, 29–34. 27868085 10.1016/j.copsyc.2016.10.004 PMC5111863

[bibr49-0306624X241252052] Merton R. K. Merton R. C. (1968). Social theory and social structure. Simon and Schuster.

[bibr50-0306624X241252052] Molleman T. Van Ginneken E. (2015). A multilevel analysis of the relationship between cell sharing, staff–prisoner relationships, and prisoners’ perceptions of prison quality. International Journal of Offender Therapy and Comparative Criminology, 59(10), 1029–1046. 10.1177/0306624X14525912 24618876

[bibr51-0306624X241252052] Moore T. McArthur M. Death J. (2020). Brutal bullies and protective peers: How young people help or hinder each other’s safety in residential care. Residential treatment for children & youth, 37(2), 108–135. 10.1080/0886571X.2019.1682487

[bibr52-0306624X241252052] Moore T. McArthur M. Death J. Tilbury C. Roche S. (2017). Young people’s views on safety and preventing abuse and harm in residential care: “It’s got to be better than home”. Children and Youth Services Review, 81, 212–219. 10.1016/j.childyouth.2017.08.010

[bibr53-0306624X241252052] Moos R.H. (1975). Evaluating correctional and community institutions. Wiley-Interscience.

[bibr54-0306624X241252052] Moos R. H. Houts P. S. (1968). Assessment of the social atmospheres of psychiatric wards. Journal of Abnormal Psychology, 73(6), 595–604. 10.1037/h0026600 5717365

[bibr55-0306624X241252052] *Moos R. H. Houts P. S. (1970). Differential effects of the social atmosphere of psychiatric wards. Human Relations, 23(1), 47–60. 10.1177/001872677002300106

[bibr56-0306624X241252052] *Neimeijer E. G. Delforterie M. J. Roest J. J. Van der Helm P. Didden R. (2021). Group climate, aggressive incidents and coercion in a secure forensic setting for individuals with mild intellectual disability or borderline intellectual functioning: A multilevel study. Journal of Applied Research in Intellectual Disabilities, 34, 1026–1036. 10.1111/jar.12841 33305516 PMC8359421

[bibr57-0306624X241252052] Niv S. Tuvblad C. Raine A. Baker L. (2013). Aggression and rule-breaking: Heritability and stability of antisocial behavior problems in childhood and adolescence. Journal of Criminal Justice, 41(5), 285–291. 10.1016/j.jcrimjus.2013.06.014 PMC3856338 24347737

[bibr58-0306624X241252052] O’Donnell I. Edgar K. (1999) Fear in prison. The Prison Journal, 79, 90–99. 10.1177/0032885599079001006

[bibr59-0306624X241252052] Page M. J. McKenzie J. E. Bossuyt P. M. Boutron I. Hoffmann T. C. Mulrow C. D. Shamseer L. Tetzlaff J. M. Akl E. A. Brennan S. E. Chou R. (2021). The PRISMA 2020 statement: An updated guideline for reporting systematic reviews. BMJ, 372, 71. 10.1136/bmj.n71 PMC8005924 33782057

[bibr60-0306624X241252052] Pappas L. N. Dent A. L. (2021). The 40-year debate: A meta-review on what works for juvenile offenders. Journal of Experimental Criminology, 19, 1–30. 10.1007/s11292-021-09472-z 34149334 PMC8196268

[bibr61-0306624X241252052] Parhar K. K. Wormith J. S. Derkzen D. M. Beauregard A. M. (2008). Offender coercion in treatment: A meta-analysis of effectiveness. Criminal Justice and Behavior, 35(9), 1109–1135. 10.1177/0093854808320169

[bibr62-0306624X241252052] Persson M. Belfrage H. Kristiansson M. (2017). Violent victimization and health service utilization in a forensic psychiatric context: A comparison between offenders with mental disorders and matched controls. BMC Psychiatry, 17, 1–10. 10.1186/s12888-017-1251-0 28284208 PMC5346204

[bibr63-0306624X241252052] Polsky H. Claster D. Goldberg C. (1968). The dynamics of residential treatment: A social system analysis. University of North Carolina Press.

[bibr64-0306624X241252052] Pompoco A. Wooldredge J. Lugo M. Sullivan C. Latessa E. J. (2017). Reducing inmate misconduct and prison returns with facility education programs. Criminology & Public Policy, 16(2), 515–547. 10.1111/1745-9133.12290

[bibr65-0306624X241252052] Pinchover S. Attar-Schwartz S. (2014). Institutional social climate and adjustment difficulties of adolescents in residential care: The mediating role of victimization by peers. Children and Youth Services Review, 44, 393–399. 10.1016/j.childyouth.2014.07.005

[bibr66-0306624X241252052] *Puzzo I. Aldridge-Waddon L. Bush E. Farr C. (2018). The relationship between ward social climate, ward sense of community, and incidents of disruptive behavior: A study of a high secure psychiatric sample. International Journal of Forensic Mental Health, 18(2), 1–11. 10.1080/14999013.2018.1532972

[bibr67-0306624X241252052] *Reisig M. Mesko G. (2009). Procedural justice, legitimacy, and prisoner misconduct. Psychology, Crime & Law, 15(1), 41–59. 10.1080/10683160802089768

[bibr68-0306624X241252052] *Robinson J. E. Craig L. (2019). Social climate and aggression in IDD services. Journal of Intellectual Disabilities and Offending Behaviour, 10(1), 8–18. 10.1108/JIDOB-11-2018-0013

[bibr69-0306624X241252052] Robinson J. E. Craig L. A. Tonkin M. (2018). Perceptions of social climate and aggressive behavior in forensic services: A systematic review. Trauma, Violence, & Abuse, 19(4), 391–405. 10.1177/1524838016663936 27519992

[bibr70-0306624X241252052] *Ros N. Van Der Helm G. H. P. Wissink I. B. Stams G. J. J. M. Schaftenaar P. (2013). Institutional climate and aggression in a secure psychiatric institution. The Journal of Forensic Psychiatry & Psychology, 24(6), 1–15. 10.1080/14789949.2013.848460

[bibr71-0306624X241252052] Rosenthal R. (1979). The file drawer problem and tolerance for null results. Psychological Bulletin, 86(3), 638. 10.1037/0033-2909.86.3.638

[bibr72-0306624X241252052] Ross M. W. Diamond P. M. Liebling A. Saylor W. G. (2008). Measurement of prison social climate: A comparison of an Inmate measure in England and the USA. Punishment & Society, 10, 447. 10.1177/1462474508095320

[bibr73-0306624X241252052] Ryan R. M. Deci E. L. (2000). Self-determination theory and the facilitation of intrinsic motivation, social development and well-being. American Psychologist, 55(1), 68–78. 10.1037/0003-066X.55.1.68 11392867

[bibr74-0306624X241252052] Schaftenaar P. Van Outheusden I. Stams G. J. J. M. Baart A. (2018). Relational caring and contact after treatment. An evaluation study on criminal recidivism. International Journal of Law and Psychiatry, 60, 45–50. 10.1016/j.ijlp.2018.07.011 30217330

[bibr75-0306624X241252052] *Schalast N. Redies M. Collins M. Stacey J. Howells K. (2008). EssenCES, a short questionnaire for assessing the social climate of forensic psychiatric wards. Criminal Behaviour and Mental Health, 18(1), 49–58. 10.1002/cbm.677. 18229876

[bibr76-0306624X241252052] *Scholte E. Van Der Ploeg J. (2000). Exploring factors governing successful residential treatment of youngsters with serious behavioural difficulties: Findings from a longitudinal study in Holland. Childhood, 7(2), 129–153. 10.1177/0907568200007002002

[bibr77-0306624X241252052] *Schubert C. Mulvey E. Loughran T. Losoya S. (2012). Perceptions of institutional experience and community outcomes for serious adolescent offenders. Criminal Justice and Behavior, 39(1), 71–93. 10.1177/0093854811426710

[bibr78-0306624X241252052] Spruit A. Van der Put C. Gubbels J. Bindels A. (2017). Age differences in the severity, impact and relative importance of dynamic risk factors for recidivism. Journal of Criminal Justice, 50, 69–77. 10.1016/j.jcrimjus.2017.04.006

[bibr79-0306624X241252052] Stoff D. M. Breiling J. Maser J. D. (1997). Handbook of antisocial behavior. Wiley.

[bibr80-0306624X241252052] Strijbosch E. L. L. Huijs J. A. M. Stams G. J. J. M. Wissink I. B. Van der Helm G. H. P. De Swart J. J. W. Van der Veen Z. (2015). The outcome of institutional youth care compared to non-institutional youth care for children of primary school age and early adolescence: A multilevel meta-analysis. Children and Youth Services Review, 58, 208–218. 10.1016/j.childyouth.2015.09.018

[bibr81-0306624X241252052] Tabachnik B. G. Fidell L. S. (2013). Using multivariate statistics (6th ed.). Allyn and Bacon.

[bibr82-0306624X241252052] Tonkin M. (2016). A review of questionnaire measures for assessing the social climate in prisons and forensic psychiatric hospitals. International Journal of Offender Therapy and Comparative Criminology, 60(12), 1376–1405. 10.1177/0306624X15578834 25850103

[bibr83-0306624X241252052] *Tonkin M. Howells K. Ferguson E. Clark A. Newberry M. Schalast N. (2012). Lost in translation? Psychometric properties and construct validity of the English Essen Climate Evaluation Schema (EssenCES) Social Climate questionnaire. Psychological Assessment, 24(3), 573–580. 10.1037/a0026267 22082034

[bibr84-0306624X241252052] Tremblay R. E. (2010). Developmental origins of disruptive behaviour problems: The ‘original sin’ hypothesis, epigenetics and their consequences for prevention. Journal of Child Psychology and Psychiatry, 51(4), 341–367. 10.1111/j.1469-7610.2010.02211.x. 20146751

[bibr85-0306624X241252052] Ungar M. (2013). Resilience, trauma, context and culture. Trauma, Violence, & Abuse, 14(3), 253–264. 10.1177/1524838013487805 23645297

[bibr86-0306624X241252052] Van Dam L. Smit D. Wildschut B. Branje S. Rhodes J. Assink M. Stams G. J. J. M . (2018). Does natural mentoring matter? A multilevel meta-analysis on the association between natural mentoring and youth outcomes. American Journal of Community Psychology, 62(1–2), 203–220. 10.1002/ajcp.12248 29691865 PMC6174947

[bibr87-0306624X241252052] Van den Noortgate W. López-López J. A. Marín-Martínez F. Sánchez-Meca J . (2014). Meta-analysis of multiple outcomes: A multilevel approach. Behavior Research Methods, 47, 1274–1294. 10.3758/s13428-014-0527-2 25361866

[bibr88-0306624X241252052] *Van den Tillaart J. Eltink E. Stams G. J. J. M. Van der Helm P. H. Wissink I. B . (2018). Aggressive incidents in residential youth care. International Journal of Offender Therapy and Comparative Criminology, 62(13), 3991–4007. 10.1177/0306624X18758898 29490532 PMC6136074

[bibr89-0306624X241252052] Van der Helm G. H. P. Beunk L. Stams G. J. J. M. Van der Laan P. H. (2014). The relation between detention length, living group climate, coping and treatment motivation among juvenile delinquents in a youth correctional facility. The Prison Journal, 94, 260–275. 10.1177/0032885514524884

[bibr90-0306624X241252052] Van der Helm G. H. P. Boekee I. Stams G. J. J. M. Van der Laan P. H. (2011). Fear is the key: Keeping the balance between flexibility and control in a Dutch youth prison. Journal of Children’s Services, 6(4), 248–263. 10.1108/17466661111190947

[bibr91-0306624X241252052] Van der Helm G. H. P. Kuiper C. H. Z. Stams G. J. J. M . (2018). Group climate and treatment motivation in secure residential and forensic youth care from the perspective of self- determination theory. Children and Youth Services Review, 93, 339–344. 10.1016/j.childyouth.2018.07.028

[bibr92-0306624X241252052] *Van der Helm G. H. P. Matthys W. Moonen X. Giesen N. Van der Heide E. Stams G. J. J. M . (2013). Measuring inappropriate responses of adolescents to problematic social situations in secure institutional and correctional youth care: A validation study of the TOPS-A. Journal of Interpersonal Violence, 28(8), 1579–1595. 10.1177/0886260512468322 23266998

[bibr93-0306624X241252052] Van der Helm G. H. P. Stams G. J. J. M. Van der Laan P. H . (2011). Measuring group climate in prison. The Prison Journal, 91(2), 158-176. 10.1177/0032885511403595

[bibr94-0306624X241252052] Van der Helm G. H. P. Stams G. J. J. M. Van der Stel J. C. Van Langen M. M. Van der Laan P. H . (2012). Group climate and empathy in a sample of incarcerated boys. International Journal of Offender Therapy and Comparative Criminology, 56, 1149–1160. 10.1177/0306624X11421649 21908495

[bibr95-0306624X241252052] *Van der Helm G. H. P. Stams G. J. J. M. Van Genabeek M. Van der Laan P. H . (2012). Group climate, personality, and self-reported aggression in incarcerated male youth. The Journal of Forensic Psychiatry & Psychology, 23(1), 23–39. 10.1080/14789949.2011.633615

[bibr96-0306624X241252052] *Van der Laan A. Eichelsheim V . (2013). Juvenile adaptation to imprisonment: Feelings of safety, autonomy and well-being, and behaviour in prison. European Journal of Criminology, 10(4), 424–443. 10.1177/1477370812473530

[bibr97-0306624X241252052] Van Gink R. Vermeiren N. Goddard L. van Domburgh B. van der Stegen J. Twisk A. Popma L. J. (2018). The influence of Non-violent Resistance on work climate, living group climate and aggression in child and adolescent residential care. Children and Youth Services Review, 94, 456–465. 10.1016/j.childyouth.2018.08.009

[bibr98-0306624X241252052] Van IJzendoorn M. H. Bakermans-Kranenburg M. J. (2015). Genetic differential susceptibility on trial: Meta-analytic support from randomized controlled experiments. Development and Psychopathology, 27, 151–162. 10.1017/s0954579414001369 25640837

[bibr99-0306624X241252052] *Van Wijk-Herbrink M. F. Arntz A. Broers N. J. Roelofs J. Bernstein D. P. (2021). A schema therapy based milieu in secure residential youth care: Effects on aggression, group climate, repressive staff interventions, and team functioning. Residential Treatment for Children and Youth, 38(2), 289–306. 10.1080/0886571X.2019.1692758

[bibr100-0306624X241252052] Viechtbauer W. (2010). Conducting a meta-analysis in R with the metafor package. Journal of Statistical Software, 36, 1–48. 10.18637/jss.v036.i03

[bibr101-0306624X241252052] Viechtbauer W. (2015). Meta-analysis package for R. https://cran.rproject.org/web/packages/metafor/metafor.pdf

[bibr102-0306624X241252052] White L. M. Aalsma M. C. Salyers M. P. Hershberger A. R. Anderson V. R. Schwartz K. Dir A. L. McGrew J. H. (2019). Behavioral health service utilization among detained adolescents: A Meta-analysis of prevalence and potential moderators. Journal of Adolescent Health, 64(6), 700–708. 10.1016/j.jadohealth.2019.02.010 31122506

[bibr103-0306624X241252052] Whittaker J. K. Del Valle J. F. Holmes L. (2015). Therapeutic residential care for children and youth: Developing evidence-based international practice. Jessica Kingsley Publishers.

[bibr104-0306624X241252052] Willis J. Willis M. (2020). Based Strategies to ignite student learning: Insights from neuroscience and the classroom. ASCD.

[bibr105-0306624X241252052] Wright K. N. (1985). Developing the prison environment inventory. Journal of Research in Crime and Delinquency 22(3), 257–277.

[bibr106-0306624X241252052] Yoon I. A. Slade K. Fazel S. (2017). Outcomes of psychological therapies for prisoners with mental health problems: A systematic review and meta-analysis. Journal of Consulting and Clinical Psychology, 85(8), 783–802. 10.1037/ccp0000214 28569518 PMC5518650

