# Supplementary material for: Safety First! Residential Group Climate and Antisocial Behavior: A Multilevel Meta-analysis
Source: Int J Offender Ther Comp Criminol. 2024 Jun 10;69(12):1663–87. doi: 10.1177/0306624X241252052 (PMC12287560; doi:10.1177/0306624X241252052)
Supplement: sj-docx-1-ijo-10.1177_0306624X241252052 – Supplemental material for Safety First! Residential Group Climate and Antisocial Behavior: A Multilevel Meta-analysis [file sj-docx-1-ijo-10.1177_0306624X241252052.docx]

**Appendix I**

Database: Ovid MEDLINE(R) ALL <1946 to January 30, 2023>, ERIC <1965 to October 2022>, APA PsycInfo <1806 to January Week 4 2023>

Search Strategy:

--------------------------------------------------------------------------------

1 ("Ward Atmosphere Scale" or "Correctional Institutions Environment Scale" or "Prison Environment Inventory" or "Prison Preference Inventory" or "Prison Social Climate Survey" or "Measuring the Quality of Prison Life" or "Quality of Prison Life questionnaire" or "Essen Climate Evaluation Scale" or "Prison Group Climate Instrument" or "Styve" or "staff quality of life" or "Essen Climate Evaluation").mp. [mp=ti, bt, ab, ot, nm, hw, fx, kf, ox, px, rx, ui, an, sy, id, tc, tm, mf] (462)

2 ("ward climate" or "group climate" or "residential climate" or "institutional climate" or "group atmosphere" or "living climate" or "therapeutical climate" or "therapeutic* atmosphere" or "person environment interaction" or "psychiatric ward*" or "social climate" or "group environment" or "ward atmosphere" or "work climate").mp. [mp=ti, bt, ab, ot, nm, hw, fx, kf, ox, px, rx, ui, an, sy, id, tc, tm, mf] (11234)

3 1 or 2 (11310)

4 (aggress* or external* or delinq* or criminal* or offen* or recidiv* or conduct or anger or oppositional or violen* or defiant* or incident* or antisocial or reoffen* or conflict* or fighting or "behavior problems" or "behaviour problems" or misconduct or assault or disruptive or disturbance or "behav* difficulties").mp. [mp=ti, bt, ab, ot, nm, hw, fx, kf, ox, px, rx, ui, an, sy, id, tc, tm, mf] (2286763)

5 3 and 4 (2736)

6 remove duplicates from 5 (2201)

7 6 (2201)

8 limit 7 to all journals [Limit not valid in Ovid MEDLINE(R),Ovid MEDLINE(R) Daily Update,Ovid MEDLINE(R) PubMed not MEDLINE,Ovid MEDLINE(R) In-Process,Ovid MEDLINE(R) Publisher,ERIC; records were retained] (1920)

9 limit 8 to english language (1599)

10 (school or university).mp. [mp=ti, bt, ab, ot, nm, hw, fx, kf, ox, px, rx, ui, an, sy, id, tc, tm, mf] (2058917)

11 9 not 10 (1381)

12 11 not covid*.mp. [mp=ti, bt, ab, ot, nm, hw, fx, kf, ox, px, rx, ui, an, sy, id, tc, tm, mf] (1369)

13 12 not elderly.mp. [mp=ti, bt, ab, ot, nm, hw, fx, kf, ox, px, rx, ui, an, sy, id, tc, tm, mf] (1354)

14 (animal* or dog* or mice).mp. [mp=ti, bt, ab, ot, nm, hw, fx, kf, ox, px, rx, ui, an, sy, id, tc, tm, mf] (8049184)

15 13 not 14 (1345)

16 15 not sport*.mp. [mp=ti, bt, ab, ot, nm, hw, fx, kf, ox, px, rx, ui, an, sy, id, tc, tm, mf] (1320)

17 (somatic* or cancer or medication).mp. [mp=ti, bt, ab, ot, nm, hw, fx, kf, ox, px, rx, ui, an, sy, id, tc, tm, mf] (2703411)

18 16 not 17 (1225)

19 (experiment* or RCT or "control group" or "comparison group" or "case study" or "case report" or SCED).mp. [mp=ti, bt, ab, ot, nm, hw, fx, kf, ox, px, rx, ui, an, sy, id, tc, tm, mf] (4495793)

20 18 not 19 (1117)

21 20 not burnout.mp. [mp=ti, bt, ab, ot, nm, hw, fx, kf, ox, px, rx, ui, an, sy, id, tc, tm, mf] (1084)

22 (organizational or organisational).mp. [mp=ti, bt, ab, ot, nm, hw, fx, kf, ox, px, rx, ui, an, sy, id, tc, tm, mf] (408826)

23 21 not 22 (982)

24 23 not IPV.mp. [mp=ti, bt, ab, ot, nm, hw, fx, kf, ox, px, rx, ui, an, sy, id, tc, tm, mf] (979)

25 (geriatric* or dement*).mp. [mp=ti, bt, ab, ot, nm, hw, fx, kf, ox, px, rx, ui, an, sy, id, tc, tm, mf] (394392)

26 24 not 25 (961)

27 (diabet* or smok* or pharm*).mp. [mp=ti, bt, ab, ot, nm, hw, fx, kf, ox, px, rx, ui, an, sy, id, tc, tm, mf] (5506649)

28 26 not 27 (934)

***************************
